# Clinical Characteristics of COVID-19 Related Deaths in Ethiopia

**DOI:** 10.4314/ejhs.v31i2.3

**Published:** 2021-03

**Authors:** Menbeu Sultan, Desalegn Kene, Woldesenbet Waganew, Aschalew Worku, Aklilu Azazh, Beza Girma, Yakob Seman, Natinael Tessema, Sisay Yifru, Sisay Teklu, Rahel Argaw, Muluwork Tefera, Miraf Walelegn, Berhane Redae

**Affiliations:** 1 St. Paul's hospital millennium medical college, Addis Ababa, Ethiopia; 2 University of Gondar, college of medicine and health sciences, Gondar Ethiopia; 3 Addis Ababa University College of health science. Addis Ababa, Ethiopia; 4 Federal ministry of health Ethiopia, Addis Ababa, Ethiopia; 5 Save the children Ethiopia, Addis Ababa, Ethiopia

**Keywords:** Mortality, COVID-19, Ethiopia

## Abstract

**Background:**

Since the occurrence of COVID-19 in the world, it has claimed nearly 1.39 million human lives in the world and more than 1500 lives in Ethiopia. The number of deaths is increasing with variable distribution in the world. Despite its increasing fatality, the clinical characteristics of the deceased patients are not yet fully known. Analyzing the clinical characteristics of deceased patients will help to improve the outcome of infected patients. Hence, this study aimed to determine the clinical characteristics of patients who died due to COVID-19 in Ethiopia.

**Methods:**

Hospital based multi-center cross-sectional study was conducted using chart review of deceased patients. Since the number of COVID-19 related deaths was limited, all consecutive COVID-19 related hospital deaths were analyzed. The data was entered into and analyzed using SPSS version 25.0. Descriptive statistics was used to explain the data collected from the survey.

**Result:**

A total of 92 deceased patient charts were analyzed. Of these patients, 65(71%) were males. Age ranged from 17 to 92 years (mean age being 59 years). On arrival vital signs, 60.5% of them had hypoxia, 49% had tachycardia and only 32% of patients had fever. Three fourth of the patients 64/85 had at least one comorbidity. Diabetes mellitus (DM) was the commonest comorbidity accounting for 445.9%, followed by hypertension, 23/85(27%), and HIV/ AIDS, 15/85 (17.5%).

**Conclusion:**

The results of this study showed that COVID-19 deceased patients presented with respiratory failure and hypoxia. However, less than a third of these patients had fever. In addition, the presence of comorbid illnesses and non-COVID-19 diseases like AIDS defining illness in significant amount needs further study to identify their level of contribution to the increasing burden of COVID-19 deaths in Ethiopia.

## Introduction

Acute respiratory infection detected in China at the end of 2019 has been described as COVID-19 by the World Health Organization (WHO)(1). The virus causing this disease is related to SARS virus and is described as Severe Acute Respiratory Syndrome Corona virus 2 (SARS CoV2), by the International Committee on Taxonomy of Viruses(2). It can be transmitted from person to person through respiratory droplets, contact and aerosols (3). Due to the rapid spread of COVID-19, lethality in severe cases, and no specific medicine, it poses a huge threat to human life and health. The World Health Organization (WHO) declared COVID-19 outbreak as the sixth public health emergency of international concern and also declared the outbreak as pandemic on 30^th^ of January and 11^th^ of March, 2020 respectively (3).

COVID-19 is highly contagious affecting more than 58 million people worldwide (4). As of this article writing, COVID-19 related death is nearly 1.39 million in the world and more than 1500 in Ethiopia (4). There is no agreed clear definition regarding deaths due to COVID-19 in the international reports so far. Some countries defined COVID-19 related mortality as a death in patients who test positive for SARS-CoV 2, independently from pre-existing diseases that may have caused death. The World Health Organization (WHO) defined death resulting from a clinically compatible illness, in a probable or confirmed COVID-19 case, unless there is a clear alternative cause of death that cannot be related to COVID -19 (e.g. trauma) (5).

There is increasing evidence that people with existing chronic conditions or compromised immune systems are at higher risk of death from COVID-19. As it is reported from deaths in China describing the clinical characteristics of 82 deaths, a total of 17.1% were treated in the ICU, 83% of deaths never received Critical Care Support. In another study, only 40% had mechanical ventilation support despite 100% needed oxygen and the leading cause of death being pulmonary related disease (6,7,8). Mortality from COVID-19 ranges between 2–10% and varies according to the setting, country, hospital type and other factors such as presence of comorbidities (9). In Ethiopia, patients with confirmed COVID-19 disease are cared for at designated treatment centers.

Although the above mentioned publications from the developed nations and some basic researches showed the more complicated nature of the disease, there is a scarcity of published data regarding the clinical characteristics of deaths related to COVID-19 in low and middle income countries. The unique demographic and epidemiologic factor in developing nations like Ethiopia demands the analysis of clinical characteristics of deceased patients in these centers. This will help to understand the characteristics of diseased patients and to improve the outcome of infected patients. Hence, in this cross-sectional study, we aimed to describe the demographics, risk factors and clinical course of patients who died in COVID-19 treatment centers in Ethiopia. These findings can shed light on the true burden of COVID-19 deaths in developing nations and inform system strengthening efforts in resource-poor settings. The objective of this study was to analyze clinical characteristics of COVID-19 related deaths in Ethiopia and to describe the clinical course of these patients in the hospital.

## Materials and Methods

It is a multicenter institution-based cross sectional study conducted from June 20, 2020 to August 20, 2020 in Ethiopia. Because of limited number of institutional deaths, all consecutive COVID-19 RT-PCT positive patients who died in different COVID-19 treatment centers and isolation centers are included in the study. We excluded pediatrics deaths and incomplete charts. In addition, the institutions were selected purposively based on burden of death, convenience and access of data.

Data was collected from patients' charts from COVID-19 treatment centers at hospitals in Addis Ababa, namely SPHMMC, Ekka Kotebe, St. Peter and Yekatit 12 while Borumeda Treatment Center was included from Amhara region. Few patients who died at Tikur Anbessa Specialized Hospital Isolation Center were also included. Moreover, when information in the charts reviewed was incomplete, treating physicians were interviewed.

Data collection tool, which was developed by the clinical advisory team for COVID-19 at Ministry of Health of Ethiopia, which includes demographic data, referral status, triage and severity status, clinical parameters and laboratory abnormalities, complications and course in the hospital were used for data collection. General practitioners who are working in the treatment centers were trained and used as data collectors. The data was entered and analyzed by using SPSS version 25.00 software. Descriptive statistics was used to present the data. Ethical clearance was obtained from SPHMMC institutional review board (IRB) and support letter was written from FMOH to the facilities in order to get the data.

## Results

A total of 678 patients died in Ethiopia until the reported date with COVID-19 positive result. Among these a total of 164 (24.2%) reported deaths were in the facility. Among these patients, we reviewed a total of 92 deaths. Most deaths, 65/92 (71%), were males while 27/92 (29%) were females. The mean age of the 92 dead patients was 59 years, ranging from 17 to 92 years. Most patients, 71/92 (77.2%), were referred from other facility before death. More than half of them were admitted in the referring facility before referral to the last facility. The commonest reason for referral being for COVID-19 care followed by for hemodialysis, and for better ICU care (Table 1).

**Table 1:** Reason for inter-facility transfer in COVID-19 diseased patients in Ethiopia, 2020

Reason for referral	Number N (%)
COVID-19 care	30(42%)
Dialysis	19(28%)
Patient preference	3(4%)
Continuity of care	7(10%)
Trauma care	3(4%)
ICU care	9(12%)

**Figure 1:**
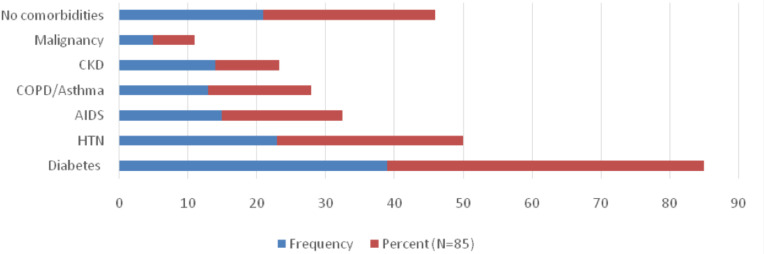
Preexisting comorbidities on COVID-19 deaths in Ethiopia, 2020

**Presenting clinical characteristics**: On arrival to the last facilities, most 57/72(79.2%) of the patients were severe to critical and the rest 15/75 (20.8%) had asymptomatic to mild illness. The commonest clinical presentations among patients were hypoxia, tachycardia and fever accounting 60.5%, 52.9% and 32% respectively. Regarding existing comorbidities, 64/85(75%) of the patients had at least one comorbidity. The commonest comorbidity was DM, 39/85 (48.9%), followed by Hypertension 23/85 (27%) and HIV 15 (17.6%). Asthma/COPD and CKD account 13/85 each and 8/85 patients respectively. Refer to Figure 1 below for more detailed information of comorbidities. Although the common diagnosis of patients during admission was respiratory condition 39/92 (42.4%), renal disease and AIDS defining illnesses were contributing for 31/92(33.4%) and 12/92(12%) respectively. At presentation, four patients were reported death on arrival to the last facility. The clinical features and the major admission diagnosis are listed in (Table 2 and Table 3).

**Table 2:** Vital signs at presentation of COVID-19 deaths in Ethiopia, 2020

Vital sign		Number	Percentage N=92)
**Fever**		29	32
**Hypothermia**		18	20
**Hypotension**		16	17.6
**Hypertensive**		19	20.6
**Tachycardia**		49	52.9
**Hypoxia**		55	60.5
**Mental status in Glasgow** **coma scale**	coma	9	10.3
	9 to 14	41	45
	conscious	41	44.8

**Table 3:** Major admission diagnosis for COVID-19 deaths in Ethiopia, 2020

Category	Diagnosis	Number	Percentage
**Respiratory (n=39)**	COVID-19 related community acquired pneumonia	23	25.0
	ARDS	**12**	13.0
	Acute exacerbation of ASTHMA/COPD	4	4.4
**Renal (n=31)**	Acute kidney injury	17	18.5
	Chronic kidney disease for dialysis	14	15.2
**Infectious (n=21)**	AIDS with opportunistic infections	12	13.0
	Sepsis/ septic shock	5	5.4
	Disseminated tuberculosis	4	4.4
**Cardio**	Congestive heart failure	6	6.5
**vascular(n=7)**	Cardiac arrest	1	1.1
**Neurologic (n=5)**	Stroke and encephalopathy	5	5.4
**Endocrine(n=4)**	Hyperglycemia complication (DKA/HHS)	4	4.4
	Electrolyte imbalance	2	2.2
	Severe anemia	1	1.1
**Surgical (n=4)**	Trauma	2	2.2
	Post-operative patient	2	2.2

**Course in the hospital**: Of all the deaths, more than half, 49/92 (53%), were admitted to ICU directly. The mean length of hospital stay before death was 7 days with a range of an hour to 17 days. More than half of these patients, 59.3%, were intubated and ventilated from one to 17 days. Of all admissions, nearly half, 45/92 (49%), of the patients were diagnosed with lethal complications after admission to hospital. As indicated in Table 4, the commonest complications diagnosed after admission were ARDS/respiratory failure (51%), Sepsis/Septic shock (44.5%) and acute renal failure (35.6%).

**Table 4:** Complications diagnosed after admission of COVID-19 deaths in Ethiopia, 2020

Parameter	Number N (%)
**ARDS/ Respiratory failure**	23(51%)
**Sepsis/septic shock**	20(44.5%)
**Coagulopathy**	7(15.6%)
**Encephalopathy/Coma**	12(27.7%)
**Venous thrombo embolism**	5(11%)
**Hospital acquired** **pneumonia**	8(17.8%)
**Acute renal failure**	16(35.6%)

## Discussion

This study is the first Ethiopian report of institutional COVID-19 related deaths. It showed that higher proportion of diseased patients were males, 65(71%), the mean age being 59 years. Almost two third of the patients had at least a visit to other non-COVID-19 treatment facility where more than half of them were admitted for a certain duration. It also showed that the majority of the deceased had comorbidities where diabetes mellitus was the commonest complication, and they had respiratory illness as a commonest complication.

In most literatures elsewhere, the median age of critically ill and deceased COVID-19 patients were older than 65 years and the majority were males. Even a higher age range of more than 80 years was reported in countries like Peru and USA (10,11,12,13). Relatively younger population of Ethiopia may explain the age discrepancy. The majority of these critically ill patients had a visit to non-COVID-19 treatment facility before referral to the last facility. While the hospitals and other facilities were using fever as screening tool for COVID-19, only 32 % of our deceased had fever on arrival to the hospital. This may have led the non-COVID treatment centers to admit these patients. Most of the deceased patients had interfacility transfer for hemodialysis. Similar to our study, in one study which evaluated inpatients deaths, kidney impairment was observed in 3.2% of patients, and those patients with kidney impairment had the higher mortality (14).

Regarding the existing comorbidities of our patients, three fourth (75%) had at least one comorbidity. The commonest comorbidity was diabetes mellitus followed by hypertension and HIV. In a report from China, similar to our finding, more than 75% of the COVID-19 deaths had comorbidities (11). However, in the same study in contrary to our study, hypertension was the commonest comorbidity found followed by cardiovascular disease. In a study on the first 25 deaths in china, the commonest comorbidities were hypertension (56.1%), heart disease (20.7%) and diabetes (18.3%), in orderly manner (10).

In a paper by Akhtar Hussain and his collogues, there is association between SARS COV 2 infection and increased prevalence of DM which is explained by inflammation, increased coagulation activity, immune response impairment, and potential direct pancreatic damage by SARS-CoV-2(15). Moreover, Diabetes was associated with increased risk for severe illness for COVID-19 and a three folds increase in mortality (16). These papers and the finding from our study indicate that there is a need to focused screening for DM, priority RT PCR test for DM patients and education focusing on these groups of patients.

The common diagnosis of the study patients during admission was respiratory condition 39/92, (42.4%). In addition, half of the patients were diagnosed with severe complications after admission. Relatively low percentage of this diagnose may be due to unavailability diagnostic methods including arterial blood gas analyzer and other laboratories. In a study elsewhere, respiratory failure remained the leading cause of death accounting for 69.5% to 100% of the deaths followed by sepsis/multi organ failure (28.0%) (17). In a study on evaluating the adherence to sepsis bundle six hours adherence is poor(8) (17,18). In our study, most patients with sepsis and septic shock were diagnosed delayed. The diagnosis of this common time sensitive COVID-19 related illness late after admission indicates the need to improve emergency and ICU quality of care.

In our study, the third comorbidity in COVID-19 related deaths was HIV AIDS, which is not reported from developed nations. In one study on HIV and risk of COVID-19 death from South Africa Western Cape Province, 16% of COVID-19 patients were HIV positive. In addition, HIV has increased the risk of COVID mortality with adjusted hazard ratio of 2.14 with confidence interval of 1.7 to 2.7. It is also reported that the focus given to HIV prevention and treatment is being decreased. Considering the increased risk of death from this patients, it is important to give due attention for these group of patients. Moreover, the presence of AIDS defining opportunistic diseases requiring ICU care, and renal disease has increased the number of organ failure, contributing significantly for patients' death. This may also indicate the need for concomitant improvement in management of non-COVID-19 critical illnesses.

Although our study is the first COVID-19 deaths report from Ethiopia and described their clinical characteristics, and important recommendations like the need to strengthen a referral system and improvement of tertiary care ICU with hemodialysis service can be taken, Generalization to the larger context is difficult due to the small samples size involved. The retrospective chart review may also be a reason for incomplete data. A future large scale study may solve this problem.
